# Improving the Functional Performance of Date Seed Protein Concentrate by High-Intensity Ultrasonic Treatment

**DOI:** 10.3390/molecules28010209

**Published:** 2022-12-26

**Authors:** Mohamed Kelany, Oktay Yemiş

**Affiliations:** 1 Food Science and Nutrition Department, Faculty of Agriculture, Sohag University, Sohag 82524, Egypt; 2 Department of Food Engineering, Faculty of Engineering, Sakarya University, Sakarya 54187, Turkey; 3 Research, Development and Application Centre (SARGEM), Sakarya University, Sakarya 54187, Turkey

**Keywords:** date seed, plant proteins, functional properties, high-intensity ultrasound, optimization

## Abstract

Date kernel is a plant-derived byproduct that has the potential to be converted into a high-value-added food ingredient, such as protein concentrate, in the food industry. Ultrasound, which is an alternative method for improving the functional properties of food proteins, is an effective physical treatment for modifying protein functionality. Solubility is the main criterion that primarily affects other functional properties of protein concentrates, such as emulsification, foaming, and water and oil binding. The aim of this study is to enhance the techno-functional performance of date seed protein concentrate (DSPC) by maximizing the solubility via a high-intensity ultrasound (HIUS) treatment at a fixed frequency of 20 kHz. The effect of ultrasonic homogenization under varying amplitudes and times (amplitude of 40, 60, and 80% for 5, 10, and 15 min, respectively) on the functional properties of the DSPC was investigated by using the response surface methodology (RSM). A face-centered central composite design (FC-CCD) revealed that the optimal process conditions of HIUS were at an amplitude of 80% for 15 min. The physicochemical and functional properties of the ultrasound-applied concentrate (DSPC-US) were determined under the optimum HIUS conditions, and then these properties of DSPC-US were compared to the native DSPC. The results showed that the solubility of all DSPC samples treated by HIUS was significantly (*p* < 0.05) higher than that of the native DSPC. In addition, emulsion activity/stability, foaming activity/stability, and oil-binding capacity increased after HIUS homogenization treatments, whereas the water-binding capacity decreased. These changes in the techno-functional properties of the DSPC-US were explained by the modification to the physicochemical structure of the DSPC (particle size, zeta potential, SDS-PAGE, SEM, FTIR, DSC, free SH content, surface hydrophobicity, and intrinsic emission). This work revealed that HIUS could be an effective treatment for enhancing the functional properties of date seed protein concentrate.

## 1. Introduction

Date palm (*Phoenix dactylifera* L.) fruit is a monocotyledonous plant that belongs to the family *Aceraceae* of the order *Arecales*, which contains 200 genera and more than 2000 species. It is the oldest fruit in the world. It has been cultivated especially in North Africa and the Middle East for millennia, although the exact origin of date palm has not been verified [1]. Date palm production around the world totals 9,454,213 tons, and the largest producer country is Egypt, producing 1,690,959 tons of date palm, which is 18% of the total date production [2]. About 11–18% of date fruit weight is the seed, which is composed of carbohydrates, dietary fiber, fat, ash, and protein [3]. Agricultural byproducts have functional ingredients that are important for the utilization and development of renewable sources to meet the protein demand of the nutrition and health industry [4]. Date seeds are considered an underutilized byproduct and a significant problem for the food industry. It is predicted that approximately 1.0–1.7 million metric tons (Mt) of date seed are generated as a byproduct per year, considering the annual production of date palm in the world and the yield of seeds. Several studies previously reported that the protein content of a date seed was about 5.56 and 5.17% [5], 2.30–6.40% [6], and 2.3–6.4 g/100 [7]. Date kernels, which can be obtained in large quantities and contain considerable amounts of protein, have the potential to be converted into value-added products through industrial processing.

Protein is one of the critical nutrients for human nutritional systems. Additionally, the quality of a protein source varies significantly depending on its bioavailability, digestibility, amino acid content, purity, anti-nutritional factors, and processing effects [8]. According to the report on the State of Food Security and Nutrition in the World 2021 from the FAO [9], approximately 3 billion people worldwide do not consume a healthy diet. The production of protein from renewable and sustainable sources is one of the hot topics in the field of food science and technology. Recently, there has been a growing demand for novel plant-based protein ingredients that can be considered alternative ingredients in food formulations. The growing trend of vegan/vegetarian diets worldwide is due to these foods being relatively cheap and the consumer perception/awareness of plant-based proteins being healthier than animal-based proteins. In addition to these reasons, the driving force behind the demand for plant-based proteins is that the ingredients are produced from renewable and sustainable sources. For all of these reasons, the food industry is seeking new protein sources that are inexpensive and have desirable techno-functional properties. Today, legumes, cereals and pseudocereals, and oilseeds are used as protein sources for the production of commercial plant-based protein ingredients [10]. Plant-derived proteins have essential functions and can be used in processes such as emulsification, foamability, and gelation in both the food and pharmaceutical industries [11]. The techno-functional performances of proteins, which affect the behavior of proteins, the quality, and the organoleptic attributes in food, play a key role in food systems during storage, processing, preparation, and consumption [12]. The potential of plant-based proteins depends on their functionality in food systems. The poor techno-functional properties of plant-based proteins are the most noticeable drawback for their industrial application as an ingredient.

The functionality of a protein is related to its molecular structure (molecular weight, shape, and amino acid sequence). Many techniques have been used to modify the functional properties of proteins for industrial applications, such as chemical, enzymatic, and physical processes [13]. However, chemical modifications can be detrimental to the nutritional value of the products and may cause adverse effects on health. The physical processes (extrusion, high-pressure, and ultrasound homogenization) are safe and can be used to obtain attractive, functional properties of proteins and tailor them for different food applications [14]. High-intensity ultrasound (HIUS) homogenization is one of the most studied physical methods for modifying protein structures. The basic principle of HIUS is based on the microbubbles generated by sound waves, which create the shear stress and cavitation force with heat and turbulence. HIUS has many advantages, such as having a wide wave frequency and being simple, cost-effective, energy-saving, and environmentally friendly [15,16]. In recent years, the effect of high-intensity ultrasound on the physicochemical properties of plant-based proteins and, in parallel, its reflection on their techno-functional properties has been intensively studied. Several authors have investigated the effect of HIUS on the physicochemical and functional properties of soy [17,18], pea [19], black bean [20], sunflower [21], faba bean [22], millet [23], quinoa [24], peanut [25], walnut [26], and hempseed [27,28] protein isolate. Although many studies have addressed the impact of HIUS on the techno-functional performances of various plant-based protein isolates, there have been no studies on the HIUS modification of techno-functional performances of date seed protein concentrates. Limited studies on date seed proteins have primarily focused on extraction methods and the physicochemical and functional properties of the protein concentrates obtained by using these methods [29,30].

The purpose of the study was to enhance the techno-functional performance of DSPC by HIUS. In this study, DSPC was first produced by the conventional method, which was alkaline extraction and then isoelectric precipitation. The effect of the independent process variables on the solubility of DSPC was investigated, and the optimum process conditions for maximizing the protein solubility using the central composite design of Response Surface Methodology (RSM) were determined. The amplitude and time of the ultrasonic treatment were the independent process variables of HIUS. After ultrasound treatment at optimal conditions, the techno-functional performance of DSPC-US was analyzed and compared to the native DSPC.

## 2. Results and Discussion

### 2.1. Proximate Chemical Composition

Table 1 shows the major components (moisture, protein, fat, ash, and total carbohydrate) of date seed, defatted date seed, and DSPC. Carbohydrates and fat form the main portion of date seed composition in terms of amount. The crude protein and fat content of date seed used in this study were 6.17% and 9.56%, respectively. There are many studies on the chemical composition of date seeds in the literature. It has been reported that the protein and fat content was in the range of 2.29–7.08% and 5.02–13.3%, respectively [5,29,31]. However, we are unable to make comparisons because no information on the protein content of the “Saidy” variety is available in the literature. The obtained results were in accordance with those reported by Besbes et al. [5], who studied the seed of “Deglet Nour” and “Allig” cultivars grown in Tunisia at the ‘tamar’ stage of maturity. They reported that the protein contents of these cultivars were 5.56% and 5.17%, respectively. These considerable differences among the cultivars in the chemical composition of date seeds could be related to many factors such as cultivar, fruit ripeness, agricultural practices, and the geographical and climatic conditions. The results revealed that the “Saidy” variety used in this study had a relatively high protein and fat content; therefore, date seed may become a potential source in terms of the conversion of a byproduct into a value-added product. The DSPC produced using the alkaline extraction–isoelectric precipitation method had a 70.28% protein content. Akasha et al. [29] conducted treatments on the conversion of defatted date seed flour to protein concentrate using five different methods. They reported that the protein powder obtained using the alkaline extraction–isoelectric precipitate method only had a protein content of 11.79%, which was much lower than the result (70.28%) shown in this study. The contradictory finding may be attributed to the extraction conditions such as time, temperature, solid: liquid ratio, and centrifugation, which could have changed the protein content of the concentrates.

### 2.2. Response Surface Modeling

Table 2 shows the process variables (amplitude and time) and protein solubility values obtained under different combinations of high-intensity ultrasound conditions by the face-centered central composite design (FC-CCD). The ‘Fit Summary’ statistics (*F*, *p* value, Lack-of-fit and, *R*^2^) produced using the Design Expert software were used to decide the best-fit model for the protein solubility. The ‘Fit Summary’ statistics indicated that the quadratic model is highly significant for protein solubility. Analysis of variance and the regression coefficients of the suggested quadratic model are given in Table 3. The ‘lack-of-fit’ value of the suggested quadratic model for protein solubility was 0.1052, confirming the fitting of the data to the proposed model (0.1052 > 0.05). The probability (*p*) values lower than 0.05 was another criterion showing that the model and variables are significant. The ANOVA analysis showed a *p*-value of 0.034 for the quadratic model, confirming the significance of the model. Both amplitude and time significantly affected the protein solubility at the 0.01 level.

(1) $$\mathrm{RD}=\left[\frac{\mathrm{actual}\;\mathrm{value}-\mathrm{predicted}\;\mathrm{value}}{\mathrm{actual}\;\mathrm{value}}\right]\times 100$$

The coefficient of determination (*R*^2^) and the adjusted *R*^2^ were considered to check the adequacy of the model. The *R*^2^ value for the suggested quadratic model was 0.9173, which means that 91% of the experimental data was compatible. As a general rule, the *R*^2^ value for good model adequacy and fitness should be greater than 0.8 [32]. A high *R*^2^ and low *p*-value revealed that the suggested quadratic model is sufficient to represent the relationship between the variables and response. The best regression equation, which is an empirical relationship between the protein solubility and the process variables of high-intensity ultrasound, was presented in terms of the coded values in Equation (2). Each of the actual responses obtained from the 12 different conditions was compared to the predicted values calculated from Equation (2) to check the adequacy of the suggested model (Table 2). The results revealed that the proposed model could be used to predict the optimal conditions for protein solubility.

Y_Solubility_ = + 22.81 + 3.23A + 2.50B − 0.30AB + 5.16A^2^ − 0.57B^2^ (2)

where A is US amplitude (%) and B is US time (min).

### 2.3. Optimization of High-Intensity Ultrasound Variables Based on the Protein Solubility

The values of DSPC solubility after the optimization trials of ultrasound treatment are presented in Table 2, which were in the range of 20.32–32.56%. The maximum solubility of 32.56% was reached using the R2 treatment with an amplitude of 80% for 15 min (60.56 W/cm^2^). The lowest solubility (20.32%) was observed in the R1 treatment with an amplitude of 40% for 5 min (9.24 W/cm^2^), which was higher than untreated DSPC solubility (14.1%). Both amplitude (A) and time (B) had a positive influence on the solubility. It can be concluded that all of the ultrasound treatments positively affected the solubility of DSPC.

**Figure 1:**
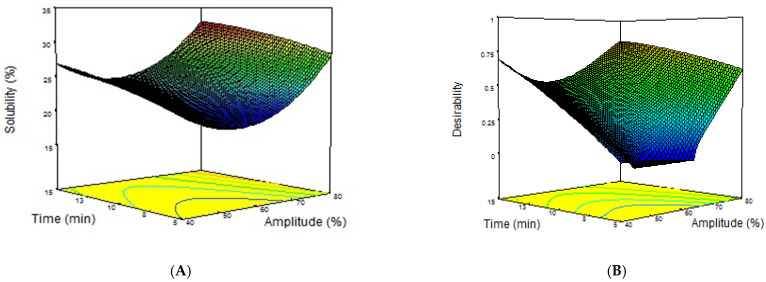
3D response figures representing the effect of HIUS amplitude and HIUS time on solubility (**A**) and desirability (**B**).

To show the main and interactive effect of the ultrasound variables on the protein solubility, the 3D response surface plots produced from Equation (2) are given in Figure 1A. The solubility of DSPC increased linearly with increasing amplitude and time in the range of 60–80% and 5–15 min, respectively. The positive effect on the protein solubility could be attributed to the increasing transferred energy into reaction media depending on the amplitude and applied time of the ultrasound. Two possible scenarios may explain this. One possible consequence is that water molecules can interact more with protein by the partial unfolding of protein molecules. Another possibility is that the ultrasound treatment can reduce the particle size of protein by the shear force and micro-streaming, which were produced by cavitation. The decreases in the particle size may increase the water–protein interaction by increasing the surface area of the protein and, therefore, enhance protein solubility. The surface response analysis for solubility revealed that the interaction between amplitude and time (AB) was non-significant (*p* = 0.7259 > 0.05, Table 3).

Our findings were in agreement with the results in the literature. The solubility of the protein isolates/concentrates could be improved by using an ultrasound treatment depending on the amplitude and time. Malik et al. [21] observed that the ultrasound treatment at a fixed acoustic power intensity of 58–61 W/cm^2^ resulted in a linear increase in the solubility of the isolate produced from sunflower meal by the treatment time up to 20 min. They reported that this linear trend disappeared after the treatment time of 20 min. Similarly, Karabulut and Yemiş [27] studied the effect of the ultrasound treatment on the functional properties of hemp seed protein isolate. They also observed a similar behavior of the isolate, in which the solubility enhanced linearly by using a treatment time of up to 10 min. They determined that the solubility decreased at ultrasound treatments at higher amplitudes of 65% and a time of 10 min. This behavior was reported in many studies on the different plant protein isolates produced from millet [23], black bean [20], faba bean [22], chickpea [33], and perilla [34]. These decreases in the solubility of the isolate after a critical energy intensity are explained by the conversion of protein molecules to higher molecular weight aggregates by non-covalent interactions. However, in our study, we did not observe decreases in the solubility of DSPC in the amplitude range of 40–80% and time range of 5–15 min. This contradictory result in our study may be explained by the fact that the intensity of energy transferred to the protein solution under the ultrasound conditions was insufficient to cause the aggregation of date seed protein molecules. Hence, the optimization studies on maximizing the solubility for the determination of the optimal ultrasound conditions are critical. Numerical optimization using Design Expert revealed that the optimal conditions of the ultrasound treatment for the maximum solubilization of DSPC were 80% amplitude for 15 min (60.56 W/cm^2^) by the highest desirability value of 0.83 (Figure 1B). The predicted optimum solubility conditions were the same as the harshest conditions (80% amplitude and 5 min) within the studied ultrasound condition ranges. The predicted solubility of DSPC under these optimal conditions by the model was 32.87%, while the actual experimental value was 32.56%, indicating that the predicted optimum value was valid. In the next stage of our study, the optimal condition consisting of an amplitude of 80% and a treatment time of 15 min was applied to the DSPC, and the techno-functional properties of DSPC-US were compared with DSPC-N.

### 2.4. Comparison of Techno-Functional Properties of DSPC

#### 2.4.1. Solubility and Water/Oil Binding

Protein solubility is the most critical techno-functional attribute due to its impacts on the performances of ingredients in food systems, especially emulsification, gelling, and foaming. It is well known that the application of protein to the food system depends on the solubility of the protein in addition to the other functional properties of protein [14]. This is the main reason for choosing solubility, which is the leading property for protein functionality, while optimizing the ultrasound variables. The samples of DSPC-US, which applied the ultrasound treatment under the optimal conditions, exhibited a 32.56% protein solubility, while the untreated ultrasound samples (DSPC-N) only had 14.1% protein solubilization (Table 4). An increase of 131% was observed in the level of solubility of DSPC after the ultrasound treatment under optimal conditions. Similarly, many studies have examined ultrasound’s effects on the solubility of protein isolates/concentrates produced from various plant materials. The solubility values for the protein isolates applied to ultrasound treatment have been previously declared for hemp seed (78% [27]), faba bean (77% [22]), sunflower (27% [21]), walnut (22% [26]), black bean (10% [20]), and millet [23]. Our findings for the solubility differ from the studies in the literature. Compared to these studies, the solubility of concentrate obtained from date seed was high for the ultrasound treatments. There are two plausible reasons for these differences in protein solubility. The first one was using different process conditions (amplitude, time, and protein concentration) for the ultrasound treatment, which means that transferred energy intensity into reaction media can differ depending on process conditions. Another possible reason was that different plant-derived proteins have different molecular structures.

The positive effect of the HIUS treatment on solubility relies on the high temperature and pressure formed by acoustic cavitation, turbulent flow, shear force, micro-streaming, and shock waves, which are responsible for chemical and physical modifications in protein molecules [35,36]. The observed increase in the solubility of DSPC using the ultrasound treatment could be attributed to the conformational and physical changes in the protein. Similarly, Martínez-Velasco et al. [22] studied the effect of high-intensity ultrasound conditions on the surface, foaming, and structural properties of faba bean protein by using the surface response methodology. However, the models were not significant for solubility, so they could not determine the optimum conditions of ultrasound. Mir et al. [37] showed that the solubility of album seed protein isolates improved by increasing residence time to up to 25 min at a fixed amplitude of 25%. They concluded that the effect of the ultrasound treatment on the solubility was due to the exposure of the hydrophilic groups and increased electrical conductivity, representing the conformational changes in the protein molecules. Similar findings were reported in various studies with different plant protein isolates, and this is explained by the partial unfolding of protein molecules in which the exposure of the buried hydrophilic groups depends on the applied ultrasound intensity and time [20,23,33]. In our study, the change in the solubility of DSPC by using the ultrasound treatment was confirmed with conformational changes, which were shown in the FTIR and zeta potential results (Table 4). The shifts in amid I and amid A regions have revealed the conformational changes of date seed protein by using the ultrasound treatment. Furthermore, the conformational changes upon the initiation of the ultrasound treatment were confirmed by the zeta potential and DSC results. However, this phenomenon was not confirmed by the SDS-PAGE findings that revealed no change in the protein profile between DSPC-N and DSPC-US. Another explanation for the increase in the solubility was the reduction in particle size when using the ultrasound treatment. It is well known that an increase in the surface area of protein leads to the interactions between protein and water [38]. The observed increase in the solubility of DSPC when using an ultrasound treatment was in agreement with the decrease in particle size, which decreased from 123 nm to 100 nm in the present study (Table 4).

The water binding capacity (WBC) and oil binding capacity (OBC) explain the interaction between water/oil and protein, which was linked to conformational characteristics, amino acid composition, and the hydrophilic and hydrophobic balance of protein [39,40]. The ability of water binding is closely related to protein solubility. As seen from Table 4, the WBC of DSPC decreased from 2.76 g/g to 1.55 g/g after ultrasound treatment, while the OBC enhanced from 1.73 g/g to 4.79 g/g. These changes indicate a 43.8% decrease in WBC and an increase of 176.8% in OBC, respectively. Similarly, Malik et al. [21] declared a similar behavior in the water and oil binding capacity of sunflower protein isolate when using an ultrasound treatment. They explained this behavior by the denaturation of proteins after ultrasound and the subsequent exposure of embedded hydrophobic groups. Recently, Karabulut and Yemiş [27] reported a similar increase in OBC by 11% for hemp seed protein isolate with a slight reduction in WHC after the HIUS treatment. The improved OBC may be due to the release of hydrophobic groups on the surface, which impact the hydrophilic–hydrophobic balance in the physicochemical solution [41]. However, we observed that the effect of the ultrasound treatment on the DSPC resulted in decreased surface hydrophobicity while the free SH content increased under optimal conditions in our study (Table 4). It can be concluded that the increase in the free SH content of DSPC paralleled the rise in OBC. In contrast to our findings, Bouaziz et al. [42] reported a WBC and OBC of approximately 4 g/g and 6 g/g for date seed protein powder produced from Allig and Deglet Nour varieties, respectively, which was much higher than the results (2.76 g/g and 1.73 g/g) presented in this study.

#### 2.4.2. Emulsifying Ability and Stability

Proteins are essential ingredients used as an emulsifier for the stabilization of food emulsions due to their amphiphilic nature. The emulsifying property characterizes the ability of a protein to be absorbed into the oil–water interface and expresses the interfacial area stabilized per unit weight of protein [43]. The stability of the emulsion is a fundamental characteristic that depends on the interactions between the oil droplets [44]. The emulsion activity index (EAI) and emulsion stability index (ESI) are used for the determination of the emulsion performance of a protein [45]. Our study showed that the EAI value of DSPC ultrasound treated at optimal conditions was 19.15 m^2^/g, while DSPC-N had an EAI of 11.92 m^2^/g. A similar trend was observed for the emulsion stability of DSPC, in which the ESI value increased from 17 min to 23 min after ultrasound treatment (Table 4). The results for the emulsion performance of DSPC have been inconsistent with the findings of Akasha et al. [30], who reported that the EAI and ESI of DSPC were approximately 50 m^2^/g and 50 min, respectively. The different results in terms of EAI and ESI could be explained by the fact that the authors used different protein extraction procedures and cultivars of date seed. Our findings indicated that the emulsifying performance of DSPC notably improved after ultrasound treatment under optimal conditions. Additionally, Kresic et al. [46] demonstrated that ultrasonic homogenization could improve the functional properties of proteins with slight changes in secondary structure. Similar studies on the effect of ultrasonic homogenization on emulsion performance have been previously conducted for various plant-based protein isolates obtained from hemp seed [27], sunflower [21], peanut [25], soy [17], millet [23], chickpea [33], and album [37]. This positive effect of ultrasound treatment on the emulsion performance of protein isolates/concentrates has been associated with the exposure of inner hydrophobic groups of protein molecules in the literature. However, our surface hydrophobicity (H_0_) results did not confirm the positive effect of ultrasound treatment, in which the H_0_ of DSPC-US was significantly lower than that of DSPC-N. Surface Hydrophobicity (H_0_) measures the number of hydrophobic groups on the surface of a protein molecule exposed in an aqueous solution [18]. In our study, the surface hydrophobicity of DSPC reduced from 164.2 to 147.3 after sonication under optimum conditions, as demonstrated in Table 4. The inconsistent result with the literature could be explained by the difference in the method of surface hydrophobicity and the material used in our study. Another plausible explanation for the decrease in the surface hydrophobicity of DSPC during ultrasound treatment may be possible oxidative reactions on the protein molecules of the free radicals generated by ultrasound waves. It is well known that free radicals occur as the result of the decomposition of water molecules by sonochemical waves [47]. Wang et al. [33] reported that the surface hydrophobicity of soy protein isolate treated with ultrasound initially reduced depending on treatment power in a range of 0–200 W and then increased with increasing power. This was explained by the oxidative aggregation that can occur as a result of covalent and non-covalent cross-linking of protein molecules in the 0–200 W range of ultrasound application. Recently, Yan et al. [48] studied the effect of flexibility and surface hydrophobicity on the emulsifying properties of ultrasound-applied soybean protein isolate. They reported that the correlation between the EAI/ESI and the flexibility was stronger than the correlation between the EAI/ESI and the surface hydrophobicity. The increase in the emulsion performance of DSPC by ultrasound treatment can be associated with the reduction in the particle size by the cavitation phenomenon, which is caused by the shear force, micro-streaming, and shock waves. The increasing solubility with the decrease in the particle size of DSPC after ultrasound application can cause proteins to diffuse and absorb more rapidly to the oil–water interface, which results in better emulsion performance. The particle size and solubility results in our study confirm this positive change in emulsion properties. Similarly, Sun et al. [49] declared that the utilization of ultrasound treatment at 20% amplitude for 20 min at emulsion conformation exhibited higher interfacial protein concentration and small droplet size, leading to improved stability against creaming. Karabulut and Yemiş [27] reported that the EAI of hemp seed protein isolates increased from 21.45 m^2^/g up to 28.14 m^2^/g when using an ultrasound treatment at an acoustic intensity of 37 W/cm^2^ for 7.8 min. Additionally, the obtained results are in accordance with the findings of Biswas and Sit [43], who reported that the emulsifying activity and stability of tamarind seed protein isolates enhanced with ultrasonication by 79.41% and 82.53%, respectively.

#### 2.4.3. Foaming Capacity and Stability

Foaming capacity (FC) and foam stability (FS) are used as indicators of the determination of the ability of foaming and permanency of the foam structure. The FC value of DSPC increased from 44% to 84% as ultrasound applied at optimal conditions, which means an increase of 91%. Similarly, the stability of the foam structure was 8% for the DSPC-N, while DSPC-US had a foam stability of 21% (Table 4). The obtained results revealed that the foaming performance of DSPC in terms of FC and FS was highly enhanced after the ultrasonic treatment under optimal conditions. The optimized condition of ultrasonic homogenization increased FC and FS by 91% and 162%, respectively. The positive effect ultrasound treatment had on the FC and FS was observed for protein isolates/concentrates obtained from sunflower [21], millet [23], hemp seed [27], faba bean [22], chickpea [33], and soy [50]. This effect of ultrasound application on the foaming performance of isolates/concentrates can be explained by the conformational and physical changes in the protein (unfolding, exposing hydrophobic groups, flexibility, denaturation temperature, particle size) as observed in the emulsion performance. Our results for the foaming properties of DSPC are confirmed by the particle size, DSC, and FTIR data. In our study, we observed that both FC and FS of DSPC improved after ultrasonication while the particle size value decreased from 123 nm to 100 nm. The enhanced solubility due to the reduction in particle size after ultrasonication can lead to increased protein adsorption at the air–water interface and stable air bubbles. Similarly, FTIR and DSC (Table 4) data revealed that the structural and thermal changes in DSPC occurred by ultrasound treatment. Recently, Karabulut and Yemiş [27] studied the modification of the functional properties of hemp seed protein isolates by using a high-intensity ultrasound treatment. They reported that the FC and FS values of 69% and 16% were improved after the ultrasound treatment under optimal conditions and explained this improvement by the increases in the surface hydrophobicity and decreases in the particle size.

### 2.5. Comparison of Physicochemical Properties of DSPC

The thermal stability of the DSPC samples was analyzed using differential scanning calorimetry (DSC), which indicated alterations in the structural and conformational of the protein during the thermal treatment. The Td and ΔH values, which are thermodynamic parameters measured using DSC analysis, indicate the denaturation temperature of the protein and the amount of heat required for denaturation, respectively. The thermodynamic parameters (Td and ΔH) obtained from the thermograms of the DSPC samples are presented in Table 4. A single endothermic denaturation peak was observed for DSPC-N and DSPC-US (data not shown). The denaturation temperature (Td) of DSPC decreased from 87.7 °C to 61.9 °C as the enthalpy value (ΔH) reduced from 204 to 191 J/g after ultrasound treatment. Our results regarding of DSPC native form are agreed with the findings of Akasha et al. [30], who worked on the Deglet Nour variety of date seeds (6% protein), and they found the denaturation temperature (Td) of 88.73 °C and the enthalpy (ΔH) of 235.6 J/g. The decreases in the Td and ΔH values revealed that the conformational changes in DSPC occurred during the ultrasound treatment, and ultrasound-treated DSPC samples can be denatured at lower energy. This change observed in the thermal stability of the DSPC samples could be attributed to the breaking intermolecular bonds of protein by the shear force formed from cavitation.

The zeta potential results of DSPC showed that the ultrasound treatment performed under optimal conditions increased the negative charge of the DSPC samples from −28.73 to −37.83 mV (Table 4). This could be attributed to the ultrasound treatment resulting in more negatively charged amino acids exposed from the inner part of the protein to the solvent. This change in zeta potential was confirmed by both FTIR and DSC data, which showed wavelength shifts at amid 1 region and a decrease in the Td and ΔH values. Similarly, Karabulut et al. [27] reported that the negative ζ potential of hemp seed protein isolates increased from −22.30 mV to −27.80 mV after ultrasound treatment. They explained that ultrasonication might generate more negatively charged residues on display from the inside part of the protein to the outside area because of unfolding protein structures. On the contrary, Xiong et al. [19] stated that ultrasound treatment on pea protein isolate had decreased the negative surface charge from −43.1 to −37.9 mV.

The particle size of the proteins has a crucial role that affects protein techno-functional properties [18]. The effect of the ultrasound treatment on the particle size of DSPC is shown in Table 4, demonstrating that the sonication treatment decreased the particle size of DSPC from 123.91 to 100.87 nm. Our results are in accordance with other studies on plant-based protein isolates/concentrates, which reveal that the sonication could decrease the particle size of proteins [18,19,38,51,52]. The reduction in the particle size of DSPC during ultrasound treatment can be ascribed to the cavitation phenomenon, which generated shear forces, micro-streaming, and shock waves [35].

**Figure 2:**
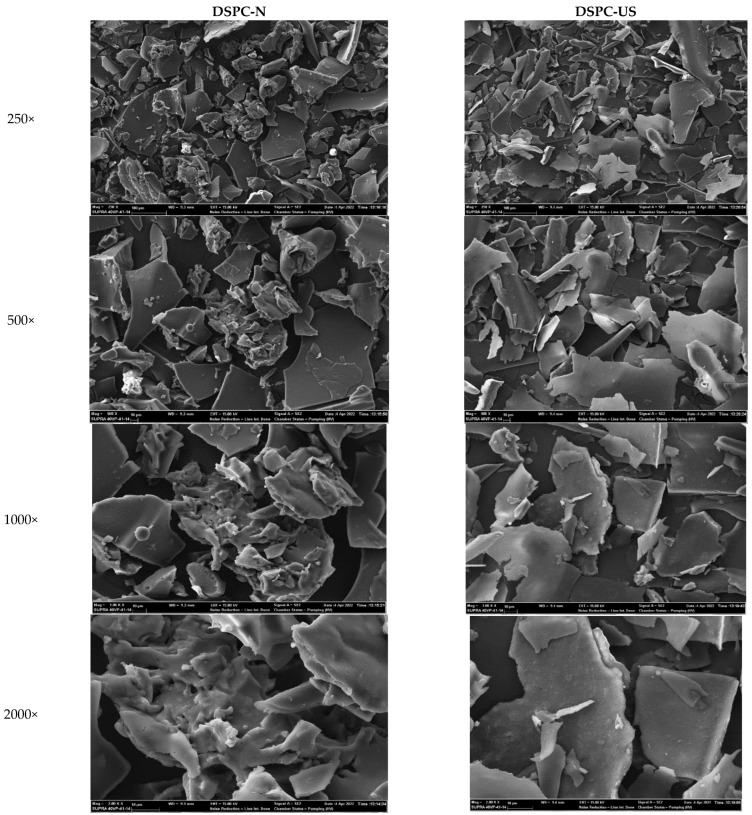
Morphology of native (DSPC-N) and ultrasound applied (DSPC-US) date seed protein concentrate determined by scanning electron microscopy.

The effect of sonication on the microstructure of DSPC was illustrated in SEM images at 250×, 500×, 1000×, 2000× magnification (Figure 2). The SEM images showed that the ultrasound-treated DSPC samples exhibited more regular fragments compared to the native form of the DSPC samples, which are in the form of clumps. These uniform structures formed by the ultrasound treatment may be associated with the unfolding of proteins and increasing free SH content (Table 4), which caused the protein interaction [53]. In our study, we observed larger aggregates, although HIUS reduced the particle size of DSPC (Figure 2). This may be explained by the form of DSPC in the analytical method used. SEM imaging was performed on the dried form of DSPC, while the particle size measurements were carried out in a liquid. It has been reported that the exposed groups by ultrasound treatment may interact during lyophilization, and form larger aggregates [18,20,54]. Similar results were recorded for canola protein isolates by Flores-Jimnez et al. [54], soy protein isolates by Hu et al. [18], and black bean protein isolates by Jiang et al. [20].

**Figure 3:**
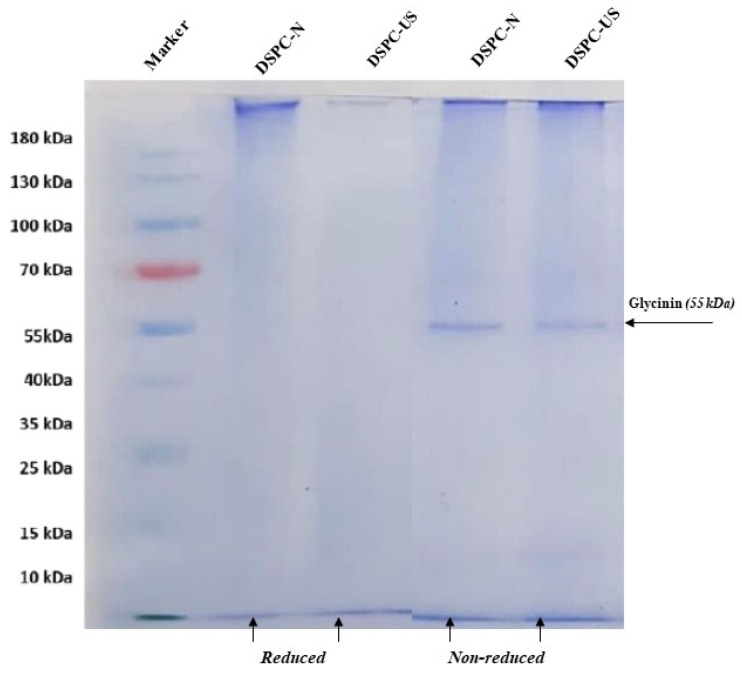
SDS-PAGE electrophoretic protein profiles of native (DSPC-N) and ultrasound applied (DSPC-US) date seed protein concentrate under reducing and non-reducing conditions.

SDS–PAGE electrophoresis was conducted to evaluate whether there was an effect of ultrasound treatment on the protein profile of DSPC. Figure 3 shows a typical SDS–PAGE profile of the DSPC samples under reducing and non-reducing conditions. Non-reducing electrophoresis revealed that the DSPC samples showed only one strong band at 55–65 kDa, which was a typical band identified as glycinin for date seed protein [30]. However, the band was not observed in SDS-PAGE under reducing conditions, indicating that the reduced fragments of protein by mercaptoethanol could be out of range of the used protein marker (10–180 kDa). Khoshroo et al. [55] studied the protein profile of twelve different date seed varieties grown in Iran. They found one heavily stained band at around 65 kDa and minor bands ranging from 12 to 369 kDa using by SDS–PAGE technique. Similarly, Bouaziz et al. [56] discovered three similar prominent protein bands at 32, 60, and 70 kDa in date seeds of Allig and Deglet Nour varieties. The variations in the protein profile of date seed could be due to the extraction method and the varieties used [57]. In our study, we did not observe any change in the number of the band between native DSPC and ultrasound-treated DSPC, but DSPC-US had a lesser band intensity than that of DSPC-N. Our results revealed that ultrasonication at the optimum conditions had no apparent effect on the primary structure of date seed proteins. Similarly, several authors reported that ultrasound treatment did not change the protein profile of various plant-based protein isolates/concentrates, such as chickpea [33], hempseed [27], and black bean [20].

**Figure 4:**
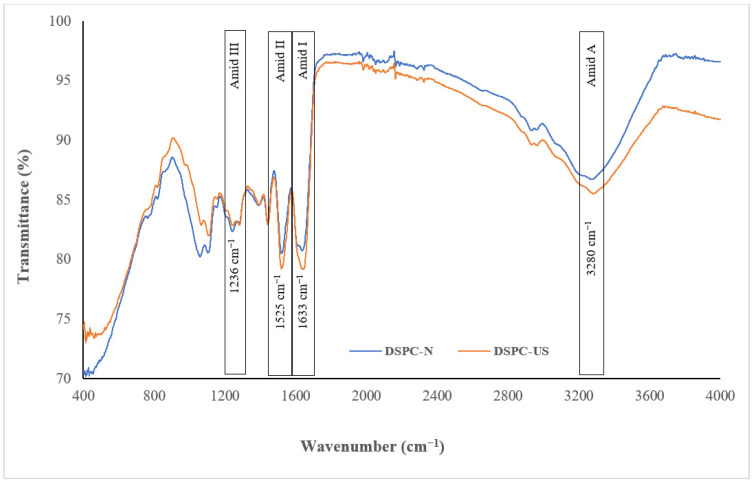
Fourier-transform infrared spectra of native (DSPC-N) and ultrasound applied (DSPC-US) date seed protein concentrate.

FTIR, a meaningful way to characterize the secondary structure of proteins by analyzing the vibrational states of the chemical bonds in the proteins, has been used to investigate the effect of ultrasound on the structure of DSPC [58]. Figure 4 shows the FTIR spectra of DSPC and US-DSPC in the range of 4000–400 cm^−1^. The DSPC samples had four typical peaks related to amid bonds, which were amid 1 (1700–1600 cm^−1^), amid 2 (1580–1480 cm^−1^), amid 3 (1400–1200 cm^−1^), amid A (3500–3200 cm^−1^). The peak of amid I shifted from 1630 cm^−1^ to 1640 cm^−1^ after ultrasound treatment, where C=O stretching and C‒N bending vibrations of the protein linkages was observed. The observed change in amid one band, which is the most sensitive to the secondary structure of the proteins, is associated with the β-sheet (1628–1642 cm^−1^) structure [59]. Moreover, we did not observe distinct shifts in amid II and amid III bands, which are located at a wavelength of 1525 cm^−1^ and 1236 cm^−1^, respectively. The prominent peak of amide A is approximately 3000–3500 cm^−1^, caused by N–H bending and O–H stretching vibrations, which are related to hydrogen bonds on the main chain of polypeptide [48]. The position of amid A shifted from 3280 cm^−1^ to 3285 cm^−1^ after ultrasonication in our study. The peaks range of amide A of DSPC and US-DSPC reported that sonication improved the hydrogen bond strength in the protein molecules of DSPC [59]. A similar result was also reported by Nazari et al. [23], who found that the peak spectra of millet protein isolate shifted from 3286 cm^−1^ to 3418 cm^−1^ after ultrasound treatment.

The content of the sulfhydryl groups on the surface of DSPC has significantly increased after HIUS from 1.58 µmol/g to 3.06 µmol/g (Table 4), which indicates the exposure of the internal SH groups of the DSPC to the surface by the cavitation effect of ultrasound [60]. Moreover, a reduction in particle size may promote the release of buried sulfhydryl groups [18]. Similarly, Karabulut et al. [27] reported that the free SH group content of hemp seed protein isolates increased from 2.65 µmol/g to 4.22 µmol/g after ultrasonication under optimal conditions. Similar results were reported for pea protein isolate by Xiong et al. [19], sunflower protein isolate by Malik et al. [21], and chickpea protein isolate by Wang et al. [33].

**Figure 5:**
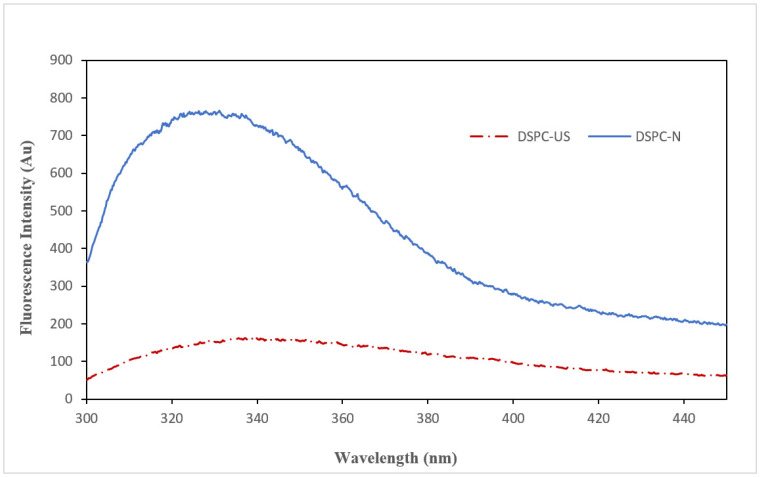
Fluorescence intensity of native (DSPC-N) and ultrasound applied (DSPC-US) date seed protein concentrate.

Intrinsic fluorescence can reflect the specific characteristics of aromatic amino acids (Tyr and Trp) in proteins and describe the tertiary structures of proteins [61]. Trp residues play a significant role in fluorescence peak wavelength from 320 to 350 nm due to the fact that the emission intensity of the Tyr residues is weak [58]. Figure 5 shows the intrinsic fluorescence spectra of DSPC-N and US-DSPC in a range of 300–450 nm. Our results showed that the maximum emission wavelength (λmax) increased from 331 nm to 339 nm while the fluorescence intensity (FI) reduced from 765 Au to 165 Au. This change observed in the fluorescence emission may be associated with the release of the chromophores in the proteins to the solvent due to the partial unfolding with ultrasound application. The decrease in FI value may be explained by the change in the environment of the protein at the molecular level after ultrasound treatment. It is well known that the molecular environment affects the fluorescence intensity of a protein. The buried chromophores inside of a protein before ultrasound treatment have a high quantum efficiency and fluorescence intensity in a hydrophobic environment. However, the molecular environment converts to a hydrophilic environment when the chromophores are exposed to water after ultrasound treatment. Thus, the quantum efficiency of the chromophores decreases by changing the molecular environment, and this conversion results in a low fluorescence intensity [62]. Liu et al. [63] reported similar results for mung bean protein hydrolysate, which decreased from 392.8 to 364.7 Au after ultrasonic treatment. Additionally, Xiong et al. [19] observed that the ultrasonic treatment (amplitude 60% and 90%) resulted in a sharp decrease (from 950 to 650 Au) in the intrinsic fluorescence intensity of ovalbumin.

## 3. Materials and Methods

### 3.1. Materials

The seeds of the Saidy variety, which is one of the most cultivated commercial date variety in Egypt, was used in this study. All of the reagents and chemicals were purchased from Merck (Darmstadt, Germany) and Sigma (Steinheim, Germany), and were of analytical grade. The distilled water used in all of the experiments was obtained from a water purification system (Elga, Purelab DV25, Lane End, UK).

### 3.2. Methods

#### 3.2.1. Production of Date Seeds Protein Concentrates (DSPC)

DSPC was produced by the conventional alkaline extraction–isoelectric precipitation method, with slight modification [29]. First, the date seeds were ground using a stone mill and passed through 40 mesh sieves. The uniform flour was defatted by using the Soxhlet extraction method for 6 h. Defatted date seed flour was air-dried at room temperature overnight and stored at 4 °C until processing. Defatted date seed flour was dispersed in distilled water at a ratio of 1:10 (solid:liquid, *w*/*v*), and then the pH of the suspension was adjusted to 10 by 0.1 M NaOH. The extraction of protein was carried out by a Heidolph heating magnetic stirrer (Schwabach, Germany) at 45 °C for 1 h. Next, the resulting slurries were spun at 10,000 rpm for 10 min at 4 °C in a centrifuge (Beckman Coulter, Allegra 64R, Brea, CA, USA). The clear supernatants containing soluble proteins were collected, and the pH value was adjusted to the isoelectric point (pH 4.5) using 0.1 *M* HCl. The mixture was centrifuged at 10,000 rpm for 10 min at 4 °C to obtain the protein pellet. The collected pellets were redissolved in an alkaline solution (pH 10) and then reprecipitated to increase the purity of the protein concentrate. The pellet was separated from the supernatant, and then the collected pellets were suspended in the minimum amount of water needed and neutralized to pH 7. The neutralized protein solutions were lyophilized in a Labconco Freezone 6 Freeze dryer (Labconco Corporation, Kansas City, MO, USA) for 72 h to produce a powder of protein concentrate. DSPC was kept in a plastic container at −20 °C until further use.

#### 3.2.2. Proximate Composition

The proximate chemical composition of the date seed, defatted date seed flour, and DSPC for moisture, protein, fat, and ash was carried out in triplicate using the methods described by AOAC [64]. The nitrogen was determined by the DUMAS method, and the nitrogen content was converted to protein by multiplying by a factor of 6.25. The carbohydrate content was calculated using the following formula (Equation (3)). All of the measurements were performed in triplicate, and the values were presented in percentages.

(3) $$\mathrm{Carbohydrate}\;\left(\%\right)=100\;–\left(\mathrm{Protein}+\mathrm{Lipid}+\mathrm{Ash}+\mathrm{Moisture}\right)$$

#### 3.2.3. High-Intensity Ultrasonic Treatment

DSPC was dissolved in water (1% *w*/*v*) at pH 7.0. The suspension was stirred at a rate of 500 rpm at room temperature for 2 h and then left overnight at 4 °C. An 80 mL of DSPC suspension in a 250 mL double-wall reactor connected to a water circulator at 10 °C (Scientz, DC2006, Ningbo, China) was ultrasonicated using a HIUS processor (Sonics, VCX750, Newtown, CT, USA) with a 1.3 cm diameter titanium probe. Each ultrasonic treatment was carried out at a constant frequency of 20 kHz under the process conditions, including amplitude (40–80%) and time (5–15 min), as outlined in Table 2. The treatment temperature was kept below about 45 °C during sonication. After the optimization study of the process conditions that maximized the solubility, the native DSPC was ultrasonicated under the optimum conditions. The processed DSPC under the optimal high-intensity ultrasound condition was defined as DSPC-US. After the HIUS treatment, the samples for the techno-functional properties (solubility, emulsification, foaming, water/oil binding) were directly analyzed, while the samples for the other analyses (zeta potential, particle size, DSC, SDS-PAGE, SEM, FTIR, surface hydrophobicity, intrinsic fluorescence emission, and free sulfhydryl group) were freeze-dried and stored +4 °C until analysis.

#### 3.2.4. Determination of Acoustic Energy

The transferred acoustic energy of the ultrasonic homogenizer probe was measured by the calorimetric method based on the measurement of the temperature increase in a liquid medium over time. In this method, the energy of the ultrasound is partly converted to the heat in the liquid medium, which results in an increase in the temperature depending on the time. The change in temperature was followed in the first 60 s of ultrasonic treatment, and a curve of temperature vs. time was created. The obtained slope (*dT*/*dt*) was used for the calculation of the delivered acoustic power. The acoustic power (W) and the ultrasound intensity (W/cm^2^) were calculated as follows in Equations (4) and (5) by following the change of suspension temperature calorimetrically during the sonication process [65]. The generated ultrasound intensities by treatment were 9.24–10.41, 22.82–24.82, and 57.62–63.29 W/cm^2^ at amplitudes of 40, 60, and 80% for 1% *w*/*v* protein concentration, respectively.

(4) $$P=m\times Cp\times \left(dT/dt\right)$$

(5) Ia= P/A

where *P* is the acoustic power (W), m is the mass of suspension (g), *Cp* is the heat capacity of the suspension (4.18 J/gK), and *dT*/*dt* is the slope of the temperature change with the time curve during the first 60 s, *Ia* is the acoustic intensity (W/cm^2^), *A* is the surface area of the HIUS probe (cm^2^).

#### 3.2.5. Response Surface Methodology

In this study, the RSM was used to determine the optimum conditions of high-intensity ultrasound treatment using the Design-Expert statistics program (version 13.0, Stat-Ease Inc., Minneapolis, MN, USA). The Face Centered Central Composite design with two variables and three levels was applied to assay the impact of amplitude A: (40, 60, and 80%) and time B: (5, 10, and 15 min) on the solubility response of DSPC. The 12 treatments, including four replicates at the center point, were run in a random order (Table 1). The results were analyzed using a quadratic model, as shown in Equation (6).

Y = β_0_ + β_1A_ + β_2B_ + β_3AB_ + β_4A²_ + β_5B²_ (6)

where Y is the estimated response variable, β_0_ is constant, β_1_, β_2_ linear, β_3_, interaction, and β_4_, β_5_ are quadratic coefficients determined by the model, while A and B are the independent variables, respectively.

The significance of the proposed models and individual model coefficients were assessed by analysis of variance (ANOVA). The validation of the proposed model for solubility was carried out by comparing the actual experimental data with predicted responses from the proposed mathematical equation. The relationships between the dependent and independent variables were investigated by the surface response plots of the polynomial regression equations. The optimal conditions based on the solubility were determined using the desirability function of the Design Expert 13.0 software.

#### 3.2.6. Techno-Functional Performances

##### Protein Solubility

The protein solubility of DSPC in water was determined using the protocol described by Morr et al. [66], with slight modifications. Ten mL of sonicated DSPC at a concentration of 1% was stirred for 1 h at room temperature after ultrasonic treatment. The suspension was centrifuged at 9000 rpm for 20 min at 25 °C, and the protein content in clear supernatant was determined by Bradford [67] method. The protein amount of DSPC (1%) in 0.1 *N* NaOH was accepted as the total protein content in DSPC. The solubility (%) was calculated using Equation (7).

(7) $$\mathrm{Protein}\;\mathrm{solubility}\left(\%\right)=\frac{\mathrm{Protein}\;\mathrm{content}\;\mathrm{in}\;\mathrm{supernatant}}{\mathrm{Total}\;\mathrm{protein}\;\mathrm{content}\;\mathrm{in}\;\mathrm{sample}}\times 100$$

##### Determination of Emulsifying Properties

The emulsion activity (EAI) and stability (ESI) indices of DSPC, which are indicators for emulsifying properties, were measured using the Spectro turbidimetric method of Pearce & Kinsella [68], with some modifications. A 3.25 mL of sunflower oil was added to 10 mL of sonicated DSPC suspension (1% in water, pH 7.0), and this mixture was homogenized at 18,000 rpm using a homogenizer (T18, Ultra-Turrax, IKA, Staufen, Germany) for 2 min. Then, 200 μL of the emulsion from the bottom of this homogenate was diluted with 25 mL of SDS solution (10 mg/mL). The absorbance (A_0_) at 500 nm was assessed to calculate EAI by a UV–Vis spectrophotometer (Shimadzu, UV-1240, Kyoto, Japan). For ESI, 200 μL of the emulsion was retaken from the bottom of the homogenate after 10 min and diluted with 25 mL of SDS solution (10 mg/mL). The absorbance (A_10_) was measured at 500 nm. Equations (8) and (9) were used to calculate the EAI and ESI, respectively.

(8) $$\mathrm{EAI}\;\left(m^{2}/g\right)=\frac{2\times T\;\times A_{0}\times \mathrm{dilution}\;\mathrm{factor}}{C\;\times \;\varphi \;\times 1000}$$

(9) $$\mathrm{ESI}\;\left(\min\right)=\frac{A_{0}}{A_{0}-A_{10}}\times \Delta t$$

where T = 2.303, A_0_ = Absorbance at zero-time, dilution factor = 100, C = the weight of protein per unit volume (g/mL), φ = the oil volumetric fraction (0.25), A_10_ = Absorbance after 10 min, Δt = 10 min.

##### Determination of Foaming Properties

The foaming capacity (FC) and stability (FS) were measured using the volumetric method described by Aydemir and Yemenicioğlu [69]. Then, 25 mL of the sonicated DSPC suspension (1%) was transferred to a 50 mL graduated Falcon conical tube. The suspension was homogenized at 20,000 rpm for 2 min by a homogenizer (T18, Ultra-Turrax, IKA, Staufen, Germany). After homogenization, the total volume of foam (V_0_) was recorded at time 0, and FC was calculated using Equation (10). The total volume of foam was remeasured after 10 min, and this value (V_10_) was used for the calculation of FS (Equation (11)).

(10) $$\mathrm{FC}\left(\%\right)=\left(\frac{\left(V0-25\right)}{25}\right)\times 100$$

(11) $$\mathrm{FS}\left(\%\right)=\left(\frac{\left(V10-25\right)}{\left(V0-25\right)}\right)\times 100$$

where V_0_ is the height of DSPC dispersion at 0 min after homogenization, V_10_ is the height after 10 min.

##### Determination of Water/Oil Binding Capacity

The water binding capacity (WBC) and oil binding capacity (OBC) of DSPC were determined according to the gravimetric method described by Aydemir and Yemenicioğlu [69], with minor modifications. A 50 mg sample of DSPC was weighed into a 2 mL Eppendorf tube, and 1 mL water or sunflower oil was added to the tubes. The suspensions were stirred by a vortex for 30 s to obtain a homogenous mixture. After incubation for 30 min at room temperature, the tubes were centrifuged at 30,000 rpm for 10 min at +5 °C. The clear supernatants were carefully decanted without material loss, and the resulting pellets were precisely weighed using an electronic balance (Ohaus, Explorer X224, Parsippany, NJ, USA). The water and oil binding capacities were determined from the differences between the weights of the samples.

#### 3.2.7. Physicochemical Properties

##### Scanning Electron Microscopy of DSPC

The morphology of dried DSPC was investigated using the scanning electron microscope of Carl Zeiss Gemini Supra 40VP field emission (Carl Zeiss SMT AG, Oberkochen, Germany) at a voltage of 15 kV. The samples were coated with platin using an ion sputter (Quorum Q 150R-ES, Quorum Technologies, Laughton, UK) and were examined in the range of 250×–2000× magnification.

##### Particle Size and Zeta (ζ) Potential Determination of DSPC

The average particle size and zeta (ζ) potential of the DSPC was determined by dynamic light scattering (DLS) using a Malvern Zetasizer Nano ZS (Malvern, Worcestershire, UK). The samples of DSPC were dissolved in distilled water (2 mg/mL) at room temperature by stirring for 1 h. The protein suspensions were centrifuged at 15,500 rpm for 10 min at +4 °C. The supernatants were filtered through Whatman No. 1 filter paper to obtain a clear filtrate. A zetasizer folded capillary cell (DTS 1070) and disposable plastic cell (DTS 0012) were used for zeta (ζ) potential and particle size analyses, respectively. The absorption and refractive index values were 0.001 and 1.330, respectively. All of the measurements were performed in triplicate, and the mean values were presented.

##### Sodium Dodecyl Sulfate-Polyacrylamide Gel Electrophoresis (SDS-PAGE)

The effect of ultrasound on the molecular structure of DSPC was evaluated by SDS-PAGE analysis, which was performed according to the method by Peng et al. [70] with slight modification. The acrylamide concentrations of the separating and stacking gels were 12% and 5%, respectively. The lyophilized DSPC samples were diluted to 1 mg/mL in the SDS-PAGE buffer by the presence or absence of 2-mercaptoethanol (β-ME). The samples were heated for 5 min at 95 °C, and then a 12 µL of protein suspension was loaded into gel lanes after cooling the samples. A commercial pre-stained protein marker at the range of 10–180 kDa (Thermo Fisher Scientific, Rockford, IL, USA) was used to identify the protein profile. The gels were stained with Coomassie Brilliant Blue R-250 and de-stained in 10% acetic acid.

##### Fourier-Transform Infrared (FTIR) Spectroscopy of DSPC

The infrared spectra of the freeze-dried DSPC samples were examined using a FTIR spectrometer (Perkin Elmer Spectrum Two, Waltham, MA, USA) with Attenuated Total Reflectance (ATR) unit. The samples were analyzed in the region of 400–4000 cm^−1^ at a resolution of 4 cm^−1^ for 16 scans at ambient conditions.

##### Surface Sulfhydryl Groups (SH) Determination

The content of free sulfhydryl groups was determined using Ellman’s reagent (5, 5′-ithiobis-(2-nitrobenzoic acid), DTNB) method, as described by Xiong et al. [19]. The DSPC samples were dissolved in Tris–HCl buffer (containing 86 mM Tris, 90 mM glycine, and 4 mM ethylenediamine tetraacetic acid (EDTA), pH 8.0) to obtain a 0.3% (*w*/*v*) protein solution. Then, 50 µL DTNB was added to the 5 mL DSPC solution. After incubation in a thermostatic shaking water bath (Witeg WSB-30, Germany) for 1 h at 25 °C, the tubes were centrifuged at 9000 rpm for 10 min at 4 °C, and the absorbances of solutions were read at 412 nm using a UV-Vis spectrophotometer (Shimadzu, UV-1240, Kyoto, Japan).

##### Intrinsic Fluorescence Emission

The intrinsic fluorescence emission spectra of DSPC (1 mg/mL in PBS buffer (10 mM, pH 7.0)) were measured at room temperature using a fluorescence spectrometer (Hitachi, F7000, Tokyo, Japan). The measurements were carried out at an excitation wavelength of 280 nm, and the emission spectra at a range of 300 nm to 450 nm using a constant 10.0 nm slit were evaluated.

##### Surface Hydrophobicity (H_0_) Determination

The surface hydrophobicity of DSPC was measured by using a fluorescence spectrum assay (Hitachi, F7000, Tokyo, Japan) using 8-anilino-1-naphthale-nesulfonicacid (ANSA) as a fluorescent probe [70]. The 10 mL DSPC solution (1 mg/mL in 10 mM PBS, pH 7.4) was mixed with 0.1 mL of the ANSA solution (2.4 mM in 10 mM PBS, pH 7.4). The excitation wavelength was 390 nm. The emission and excitation slits were 5 nm, and the emission spectrum was measured from 400 to 650 nm. The relative exposed hydrophobicity was calculated according to Equation (12).

H_0_ = S_1_ − S_2_ (12)

where S_1_ is the area of sample solution, S_2_ is the area of solvent.

##### Differential Scanning Calorimetry of DSPC

The thermal stability of DSPC was analyzed using a differential scanning calorimeter (TA Instrument Q2000 Thermal Analysis System, New Castle, DE, USA). The samples (approximately 2.5–3.0 mg) were weighed into aluminum pans, and then the pans were sealed. The sealed pans were heated from 20 to 200 °C at a rate of 5 °C/min with continuous dry nitrogen. An aluminum pan without a sample was used as a blank. TA Universal Analysis 2000 software (TA Instrument, New Castle, DE, USA) was used to compute the denaturation temperature (Td, °C) and thermal denaturation enthalpy (ΔH, J/g protein) of DSPC samples.

#### 3.2.8. Statistical Analysis

All of the experiments were carried out in triplicate. The obtained data were reported as mean ± standard deviation. The independent-sample *t*-test was used to compare the means. The one-way variance has been applied at a 95% confidence interval with the SPSS 20.0 package program (SPSS Inc., Chicago, IL, USA).

## 4. Conclusions

The main goal of the present study was to maximize the functionality of DSPC based on solubility, which is a major barrier to using DSPC. The optimum HIUS treatment conditions based on the maximum solubility were 80% amplitude and 15 min (20 kHz, 60.56 W/cm^2^), which increased the solubility by 131% compared to DSPC-N. The predicted responses agreed with the experimental responses in the used model design. After ultrasound treatment under the optimal conditions, DSPC exhibited a higher techno-functional performance, including emulsion activity/stability index, foaming capacity/stability, and oil binding capacity, except for water binding capacity. The improved techno-functional properties of DSPC by using an ultrasound treatment have been explained with the physicochemical changes, which are particle size, zeta potential, SDS-PAGE, SEM, FTIR, DSC, free SH content, surface hydrophobicity, and intrinsic emission. It can be concluded that the response surface methodology can be used to optimize the process conditions of ultrasound instead of the traditional one-factor-one-time approach. We can suggest that the optimization technique could be applied to maximize each techno-functional criteria of DSPC for specific food applications.

## Figures and Tables

**Table 1:** Principal components of date seed, defatted date seed, and DSPC as g/100 g fresh weight *.

	Moisture	Protein	Fat	Ash	Carbohydrate
Date seed	7.21 ± 0.25	6.17 ± 0.28	9.56 ± 0.14	3.44 ± 0.01	73.62 ± 0.34
Defatted date seed	6.74 ± 0.09	8.81 ± 0.21	0.49 ± 0.12	4.22 ± 0.10	79.74 ± 0.26
DSPC	5.96 ± 0.09	70.28 ± 0.33	0.13 ± 0.04	3.38 ± 0.06	20.25 ± 0.40

* Results are presented as the mean values ± standard deviation (*n* = 3).

**Table 2:** Two-factor, three level face-centered central composite design (FC-CCD) and the protein solubility of DSPC under the different conditions of high-intensity ultrasound.

Run	Process Variables			Response		
	Amplitude (%) A	Acoustic Power W/cm^2^	Time (min) B	Solubility		
				Actual	Predicted	RD * (%)
1	40 (−1)	9.24	5 (−1)	20.32	21.37	−5.2
2	80 (+1)	60.56	15 (+1)	32.56	32.87	−1.0
3	60 (0)	24.82	10 (0)	22.04	22.83	−3.6
4	60 (0)	23.52	10 (0)	22.05	22.83	−3.6
5	60 (0)	22.82	5 (−1)	20.85	19.76	5.3
6	60 (0)	24.41	10 (0)	23.38	22.83	2.4
7	80 (+1)	63.29	5 (−1)	28.36	28.47	−0.4
8	40 (−1)	10.41	10 (0)	27.02	24.75	8.4
9	60 (0)	24.18	10 (0)	21.14	22.83	−8.0
10	80 (+1)	57.62	10 (0)	31.56	31.24	1.0
11	40 (−1)	9.46	15 (+1)	25.73	26.98	−4.9
12	60 (0)	24.62	15 (+1)	26.25	24.76	5.7

* RD: relative deviation. The obtained responses were compared with the predicted responses using Equation (1).

**Table 3:** Regression coefficients and analysis of variance of the model for protein solubility of DSPC.

Variables	Solubility		
	*k*	*F*-Value	*p*-Value
Intercept	+22.81		
A-Amplitude	+3.23	23.77	0.0028
B-Time	+2.50	23.77	0.0093
AB	−0.2896	14.23	0.7259
A^2^	+5.16	0.1350	0.0020
B^2^	−0.5738	0.3324	0.5852
Lack of Fit		5.17	0.1052
Model		13.31	0.0034
R^2^		0.9173	
Adjusted R^2^		0.8484	
C.V. %		6.47	
Main effects			
Amplitude			*
Time			*

*k*, coefficients, *R*^2^, coefficient of determination, C.V., coefficient of variance, NS, not significant. * Significant at *p* < 0.05. Lack of fit is non-significant at *p* > 0.05.

**Table 4:** Techno-functional and physicochemical properties of DSPC-Native and DSPC-HIUS.

Property	DSPC-N	DSPC-US
Solubility (%)	14.10 ± 0.47 ^a^	32.56 ± 0.31 ^b^
WBC (g/g)	2.76 ± 0.18 ^a^	1.55 ± 0.17 ^b^
OBC (g/g)	1.73 ± 0.04 ^a^	4.79 ± 0.26 ^b^
EAI (m^2^/g)	11.92 ± 0.20 ^a^	19.15 ± 0.67 ^b^
ESI (min)	17.63 ± 0.36 ^a^	23.88 ± 0.53 ^b^
FC (%)	44 ± 3.82 ^a^	84 ± 2.43 ^b^
FS (%)	8 ± 0.82 ^a^	21 ± 2.43 ^b^
Td (°C)	87.7 ± 0.83 ^a^	61.96 ± 0.55 ^b^
ΔH (J/g)	204.0 ± 2.21 ^a^	191.5 ± 1.02 ^b^
Particle size (nm)	123.91 ± 1.34 ^a^	100.87 ± 1.96 ^b^
SH (µmol/g)	1.58 ± 0.17 ^a^	3.06 ± 0.24 ^b^
H_0_	164.20 ± 0.70 ^a^	147.30 ± 0.10 ^b^
(ζ) potential (mV)	−28.73 ± 1.34 ^a^	−37.83 ± 0.47 ^b^

Data are presented as mean ± standard deviation (*n* = 3). Mean values in each row with different lowercase letter superscripts are significantly different (*p* < 0.05). DSPC-N: date seed protein concentrate native form, DSPC-US: date seed protein concentrate ultrasound treated, WBO: water binding capacity, OBC: oil binding capacity, EAI: emulsion activity index, ESI: emulsion stability index, FC: foaming capacity, FS: foam stability, Td: denaturation temperature, ΔH: enthalpy, SH: free sulfhydryl group content, H_0_: surface hydrophobicity.

## Data Availability

Not applicable.
